# Administrative Restructuring Versus Product Safety: The Case for Subsequent Importer Scheme (SIS) in Importer Constitutional Changes

**DOI:** 10.7759/cureus.109281

**Published:** 2026-05-20

**Authors:** Atul Sharma Sankhyayan

**Affiliations:** 1 Operations, RAC Forge Private Limited, Thural, IND

**Keywords:** cdsco, importer constitutional change, india medtech, mdr 2017, medical device rules 2017, post-market surveillance, product safety, regulatory compliance, regulatory policy, subsequent importer scheme

## Abstract

As the Indian medical device ecosystem reaches a state of rigorous maturity, a significant friction point has emerged between corporate governance and regulatory oversight. Currently, when an established importer, an entity with an unblemished history of compliance, undergoes a routine constitutional change (such as a transition from a private limited to a limited company), the regulator often mandates a "fresh license" application. This administrative requirement effectively ignores the entity’s entire history of post-market surveillance (PMS) and safety compliance, treating a seasoned market participant as a brand-new entrant.

This article examines the "importer paradox" and argues that the logic of the Central Drugs Standard Control Organisation's (CDSCO) "Subsequent Importer Scheme" (SIS) provides a compelling blueprint for reform. We propose a shift in regulatory philosophy: prioritizing the "Safety Anchor" of the medical device, which is tethered to its technical file and manufacturing site, over the "Legal Wrapper" of the corporate entity.

By adopting a streamlined three-step transition involving a formal declaration of continuity, integration through the SIS portal, and a targeted administrative audit, the regulator can eliminate redundant filings. This approach would not only reduce the administrative burden on both the reviewer and the manufacturer but also ensure a more transparent and secure supply chain for Indian patients. Ultimately, regulatory science must recognize that corporate evolution should not be a trigger for technical re-evaluation when the underlying product safety remains unchanged.

## Editorial

As India’s medical device ecosystem matures, a paradoxical and counterproductive clash is being observed between corporate law and patient safety. Ideally, administrative rules are intended to support safety rather than complicate it. However, a major hurdle has appeared for veteran importer firms that have spent years successfully navigating the grueling licensing process of the Central Drugs Standard Control Organisation (CDSCO) under the Medical Device Rules (MDR) 2017 and the Drugs and Cosmetics Act, 1940 [1,2].

When a thriving company transitions from a private limited entity to a limited one to prepare for an Initial Public Offering (IPO) or a major merger, these transitions are recognized as hallmarks of a growing, healthy business. Yet, the current regulatory climate often views this change of "corporate skin" as a total reset. By requiring a "fresh license," a company’s entire compliance history is effectively deleted by the system. A trusted partner is treated as if they were a brand-new, unvetted stranger. This does not merely generate a mountain of paperwork; dangerous gaps are created in the market supply chain, and an administrative burden is imposed that does not actually improve patient safety.

The rollout of the "Subsequent Importer Scheme" (SIS) on the Sugam portal by the CDSCO was not just a digital update; it represented a fundamental shift in philosophy established through various notifications and administrative circulars [3]. It signaled that the regulator was prepared to cease the practice of redundant questioning. The goal was simple: to clear the path for medical devices that had already been rigorously vetted by the Central Licensing Authority.

The logic here is rooted in common sense. If a device’s safety record and clinical performance are already matters of public record, and if the product is already being used successfully by Indian doctors, there is little justification for a subsequent importer to be forced to re-initiate the process from zero. The SIS was designed to prioritize actual oversight (transparency and real-world data) over the repetitive filing of technical data that is already held in the regulator's archives [4]. It is an admission that once a product is proven safe, the "work" of that proof is considered complete.

A bizarre regulatory loop is currently being experienced. Importers with a decade-long track record of spotless post-market surveillance (PMS) and safety compliance find their history disregarded [5]. The moment a new suffix is added to a company name, the existing license is deemed worthless. In these instances, the "Legal Wrapper" is being treated as more important than the actual substance of the product.

It is maintained that extending the logic of the SIS to these constitutional changes is not just a beneficial reform; it is a logical necessity. This position rests on three core pillars: the concept of the "Safety Anchor," the problem of redundant filings, and internal logical consistency.

While the "fresh license" route may be argued as the safer bet by traditionalists, that approach is believed to be the most scientifically flawed. If a regulatory system that is truly transparent is to be achieved, the SIS framework must be adopted for constitutional changes. In practice, the "start from scratch" mentality would be replaced with a precise, three-step transition: a formal declaration of continuity, SIS-based integration, and a targeted administrative audit of the new corporate documents.

Ultimately, regulatory science should be focused on the product, not the paperwork. The "Safety Anchor" of the device must be prioritized over the legal identity of the company. By opening the SIS pathway for constitutional changes, a prime opportunity exists for the CDSCO to tear down the artificial barriers that stall corporate growth. True transparency is not found in forcing a company to rewrite its history; it is found in recognizing that history when it is already clearly established on the record.

## References

[1] Central Drugs Standard Control Organization (2017). The Medical Devices Rules, 2017.

[2] Ministry of Health and Family Welfare (1940). The Drugs and Cosmetics Act and Rules.

[3] Directorate General of Health Services (2026). CDSCO notifications and circulars. Central Drugs Standard Control Organization.

[4] Sankhyayan AS (Accessed: 22-04-2026) (2026). Administrative Restructuring vs. Product Safety—The case for SIS in importer constitutional changes. Zenodo.

[5] Sankhyayan AS (Accessed: 22-04-2026) (2026). The Evolution of Documentation Scrutiny: Internal project observations on CDSCO audit trends (2020-2026). Zenodo.

